# Evaluation of two DNA extraction methods for the PCR-based detection of eukaryotic enteric pathogens in fecal samples

**DOI:** 10.1186/s13104-018-3300-2

**Published:** 2018-03-27

**Authors:** Estelle Menu, Charles Mary, Isabelle Toga, Didier Raoult, Stéphane Ranque, Fadi Bittar

**Affiliations:** 1 Aix Marseille Univ, IRD, APHM, MEPHI, IHU-Méditerranée Infection, 19-21 Boulevard Jean Moulin, 13005 Marseille, France; 2 Aix Marseille Univ, IRD, APHM, VITROME, IHU-Méditerranée Infection, Marseille, France

**Keywords:** Enteric parasites, Protozoa, Microsporidia, qPCR, DNA extraction

## Abstract

**Objective:**

Efficient and easy-to-use DNA extraction and purification methods are critical in implementing PCR-based diagnosis of pathogens. In order to optimize the routine clinical laboratory diagnosis of eukaryotic enteric pathogens, we compare, via quantitative PCR cycle threshold (Ct) values, the efficiency of two DNA extraction kits: the semi-automated EZ1^®^ (Qiagen) and the manual QIAamp^®^ DNA Stool Mini Kit (Qiagen), on six protozoa: *Blastocystis* spp., *Cryptosporidium parvum*/*hominis*, *Cyclospora cayetanensis*, *Dientamoeba fragilis*, *Giardia intestinalis* and *Cystoisospora belli* and one microsporidia: *Enterocytozoon bieneusi*.

**Results:**

Whereas EZ1^®^ (Qiagen) and QIAamp^®^ DNA Stool Mini Kit (Qiagen) yielded similar performances for the detection of *Cryptosporidium* spp. and *D. fragilis*, significant lower Ct values (p < 0.002) pointed out a better performance of EZ1^®^ on the five remaining pathogens. DNA extraction using the semi-automated EZ1^®^ procedure was faster and as efficient as the manual procedure in the seven eukaryotic enteric pathogens tested. This procedure is suitable for DNA extraction from stools in both clinical laboratory diagnosis and epidemiological study settings.

## Introduction

Human diseases caused by eukaryotic enteric pathogens are a major public health concern [1]. Of these, protozoa are the most prevalent [2]. In order to increase the specificity and sensitivity of detection of these parasites, molecular tools have been developed over the last 10 years. PCR-based detection of eukaryotic enteric pathogens is particularly dependent upon the quality and purity of the initial DNA material. Thus, choosing an appropriate DNA extraction method is critical: it needs to (i) be highly efficient for DNA recovery from microorganisms that are frequently in oocyst form and in low abundance compared to bacterial communities and (ii) remove the many PCR inhibitors (such as bile salts, urea, hemoglobin and heparin) that are present in stools. The relatively recent introduction and rapidly increasing use of PCR-based diagnosis for eukaryotic enteric pathogen diseases requires the use of a DNA extraction procedure that should be efficient and standardized and which could be applied to the detection of all eukaryotic enteric pathogens species in order to simplify laboratory work-flow and avoid the heterogeneity of inter-laboratory results [3]. Many commercialized DNA extraction and purification kits, using chemical, enzymatic and/or mechanical lysis, are replacing in-house methods (i.e. phenol–chloroform) [4]. Studies have already been conducted to evaluate a specific combination of performance, cost-effectiveness, and simplicity of various DNA extraction kits according to the pathogen of interest [5–7], but none of these studies have directly compared these methods. In this study, we compared the performance of two commercial kits: a semi-automated EZ1^®^ procedure (Qiagen GmbH, Hilden, Germany) and a manual technique, the QIAamp^®^ DNA Stool Mini Kit (Qiagen), for the DNA extraction and purification of the most significant eukaryotic enteric pathogens [2] from stool samples.

## Main text

### Materials and methods

The comparison was carried out by direct elution of DNA from the same parasite-positive fecal samples and then by comparing the detectability of the eluted DNA by quantitative PCR (qPCR).

**Fig. 1:**
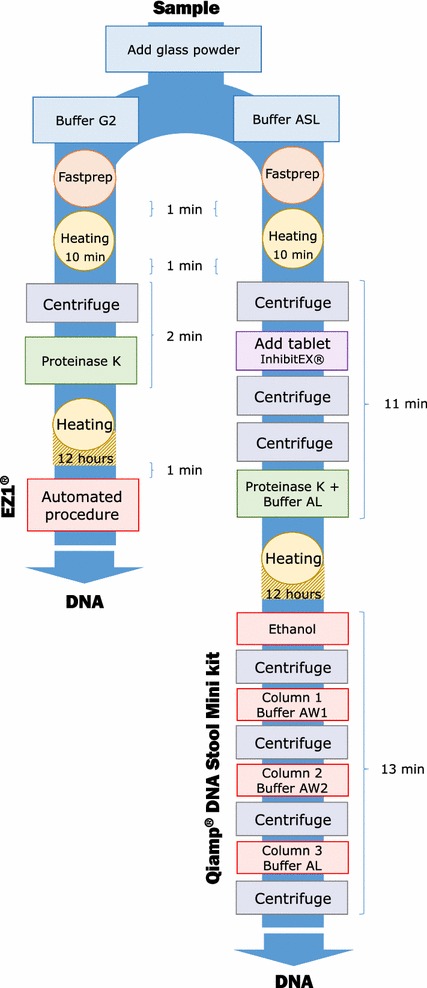
A schematic flow diagram showing the two extraction procedures: EZ1^®^ and QIAamp^®^ DNA Stool Mini Kit and time required for each step. *min* minute

This study included 24 positive fecal samples collected between January and July 2017 as part of routine laboratory diagnosis at the Parasitology–Mycology Laboratory at Marseille’s University Hospital (France). The presence of targeted eukaryotic enteric pathogens was confirmed by microscopic examination and/or routine PCR methods in our laboratory. The fecal samples were fixed in absolute ethanol and stored at 4 °C. To assess the differences in sensitivity between both extraction procedures, positive stools were serially diluted with PCR-negative stool samples. No written consent was needed for this work in accordance with the French law on bioethics (LOI No 2004-800 relative à la bioéthique) published in the “Journal Officiel de la République Française” on August 6, 2004, since no additional samples were taken for the study. According to this law, patients were informed that anonymized stool specimens could be used for further studies. A semi-automated procedure (EZ1^®^, Cat. No. 953034, Qiagen) and a manual procedure (QIAamp^®^ Stool Mini Kit, Cat. No. 51504, Qiagen) were used in parallel. For each procedure, the manufacturer’s recommendations were modified by adding mechanical, chemical and enzymatic pretreatment steps (Fig. 1). The semi-automated extraction procedure was adapted for stool processing as follows: 200 mg of stool sample was added to 350 µL of G2 lysis buffer (Qiagen) in a tube with glass powder (acid washed glass beads 425–600 µm, Sigma-Aldrich, Saint Quentin Fallavier, France) and then disrupted in a FastPrep BIO 101 apparatus (Qbiogene, Strasbourg, France) at maximum power for 40 s. After 10 min of incubation at 100 °C to allow for complete lysis, tubes were centrifuged at 10,000*g* for 1 min. Subsequently, 200 µL of supernatant was enzymatically digested using 20 µL of proteinase K (20 mg/mL, Qiagen), and incubated overnight at 56 °C. The automated procedure using the EZ1 Advanced XL extractor with the V 1.066069118 Qiagen DNA bacteria card and the EZ1^®^ DNA Tissue Kit (Qiagen, Courtaboeuf, France) was then performed for between 15 and 30 min without the intervention of an operator, as described by the manufacturer. The manual procedure, based on the use of the QIAamp^®^ Stool Mini Kit (Qiagen), which is considered as a reference method in the literature [8], was used (Fig. 1). Briefly, 200 mg of stool sample was added to 1.3 mL of ASL lysis buffer (Qiagen) in a tube with glass powder and then disrupted in a FastPrep BIO 101 apparatus at maximum power for 40 s and heated for 10 min at 95 °C. Impurities and PCR inhibitors were removed by adding inhibitEX Tablets (Qiagen) to each sample. The obtained supernatants were then enzymatically digested using 20 µL of proteinase K (20 mg/mL, Qiagen) in Buffer AL (200 µL) and incubated overnight at 56 °C. The released DNA was absorbed onto silica membranes in QIAamp mini spin columns (Qiagen). After extensive washing with AW1/AW2 Buffers, the retained DNA was eluted in AE Buffer. Nucleic acids concentration was estimated, by measuring the absorbance at 260 nm (A260), using a NanoDrop™ 2000 spectrophotometer (Thermo Scientific, Villebon-sur-Yvette, France). Then, DNA purity was assessed by determining the ratio of spectrophotometric absorbance of each extracted sample at 260 nm to that of 280 nm (A260/A280 ratio; an indicator of protein or phenol contamination). Indeed, pure DNA extraction should have an A260/A280 ratio ≥ 1.8 [5].

Total extracted DNA was used as a template for the singleplex qPCR targeting either *Blastocystis* spp., *Cryptosporidium parvum*/*hominis*, *Cyclospora cayetanensis*, *Cystoisospora belli*, *Dientamoeba fragilis*, *Enterocytozoon bieneusi*, *Giardia intestinalis* or *E. bieneusi* using specific primers and probes (Table 1), as described by Sow et al. [9]. The qPCR reactions were performed in a 20 µL total volume with 10 µL Master mix (Roche Diagnostics GmbH, Mannheim, Germany), 0.5 µL of each primer, 0.5 µL of probe, 3 µL of distilled water, 0.5 µL of Uracil-DNA glycosylase (UDG) and 5 µL of DNA. Analyses were performed using a CFX96™ Real-Time PCR detection system (BIO-RAD Life Science, Marnes-la-Coquette, France). Amplification reactions were performed as follows: 2 min at 50 °C, 5 min at 95 °C, followed by 40 cycles of 5 s at 95 °C and 30 s at 60 °C. qPCR results were considered negative when the cycle threshold (Ct) value exceeded 37 or no amplification curve was obtained, as described in previous studies [10]. To control for extraction quality and the absence of PCR inhibitors, universal eubacterial primers and probes [11] were used to amplify 16S rRNA bacterial genes, with qPCR named “all bacteria”, performed on all specimens. The statistical comparison of Ct values obtained with both extraction procedures was performed with GraphPad Prism, version 6.0 (La Jolla, CA). Normal distribution was assessed by the Kolmogorov–Smirnov test. The Student t test was used to compare the DNA extraction procedures.

**Table 1:** List of primers and probes used in this study

Organism	Name	Primers/probes	Target region
*Blastocystis* sp.	Blasto FWD F5	5′-GGTCCGGTGAACACTTTGGATTT-3′	18S
	Blasto R F2	5′-CCTACGGAAACCTTGTTACGACTTCA-3′	
	Blasto probe	5′-FAM-TCGTGTAAATCTTACCATTTAGAGGA-MGBNFQ-3′	
*Cryptosporidium parvum*/*hominis*	1PS_F	5′-AACTTTAGCTCCAGTTGAGAAAGTACTC-3′	Hsp70 gene
	1PS_R	5′-CATGGCTCTTTACCGTTAAAGAATTCC-3′	
	Crypt_P	5′-FAM-AATACGTGTAGAACCACCAACCAATACAACATC-TAMRA-3′	
*Cyclospora cayetanensis*	Cyclo250F	5′-TAGTAACCGAACGGATCGCATT-3′	18S
	Cyclo350R	5′-AATGCCACGTAGGCCAATA-3′	
	Cyclo281T	5′-FAM-CCGGCGATAGATCATTCAAGTTTCTGACC-TAMRA-3′	
*Cystoisospora belli*	Ib-40F	5′-ATATTCCCTGCAGCATGTCTGTTT-3′	ITS2
	Ib-129R	5′-CCACACGCGTATTCCAGAGA-3′	
	Ib-81Taq	5′-FAM-CAAGTTCTGCTCACGCGCTTCTGG-TAMRA-3′	
*Dientamoeba fragilis*	Df-124F	5′-CAACGGATGTCTTGGCTCTTTA-3′	18S
	Df-221R	5′-TGCATTCAAAGATCGAACTTATCAC-3′	
	Df-172revT	5′-VIC CAATTCTAGCCGCTTAT-BHQ1-3′	
*Enterocytozoon bieneusi*	FEB1	5′-CGCTGTAGTTCCTGCAGTAAACTATGCC-3′	18S
	REB1	5′-CTTGCGAGCGTACTATCCCCAGAG-3′	
	PEB1	5′-FAM-ACGTGGGCGGGAGAAATCTTTAGTGTTCGGG-TAMRA-3′	
*Giardia intestinalis*	Giardia-80F	5′-GACGGCTCAGGACAACGGTT-3′	18S
	Giardia-127R	5′-TTGCCAGCGGTGTCCG-3′	
	Giardia-105T	5′-FAM-CCCGCGGCGGTCCCTGCTAG-BHQ1-3′	
TTB (all bacteria)	TTB_16S_F	5′- AGAGTTTGATCMTGGCTCAG-3′	16S
	TTB_16S_R	5′- TTACCGCGGCKGCTGGCAC-3′	
	TTB338K_FAM	5′-FAM- CCAKACTCCTACGGGAGGCAGCAG-3′	

### Results and discussion

In both extraction methods tested, we adapted the manufacturer’s recommendations to improve DNA extraction yield by enhancing the lysis of protozoa cysts. Combining mechanical lysis using glass powder with both enzymatic and chemical lysis has been shown to significantly enhance the DNA extraction yield of eukaryotic enteric pathogens [4]. Thus, we increased the proteinase K incubation time by 12 h and added a mechanical lysis step. We objectively quantified and compared the hands-on time required to process an individual sample with each commercial kit by totaling the respective incubation times and the duration of each centrifugation step. The EZ1^®^ (Qiagen) procedure required 752 min per sample (12 h 32 min), on the other hand the QIAamp^®^ DNA Stool Mini Kit (Qiagen) procedure required 756 min (12 h 36 min). Furthermore, if we are only interested in the steps that require the operator’s intervention, the EZ1 procedure takes 15 min but this is not the case for manual technique which takes 36 min, Fig. 1. The higher the number of samples, the more complex and long the manual procedure is. We considered the EZ1^®^ (Qiagen) procedure to be superior because of its higher throughput, shorter hands-on time, and lower contamination risk, associated with fewer manual preparation steps, than with the QIAamp^®^ DNA Stool Mini Kit (Qiagen).

Then, nucleic acids yield and purity were assessed in both procedures; EZ1^®^ extraction method provided a higher concentration of nucleic acids (mean value ± standard deviation = 29.61 ± 18.46 ng/µL) and lower levels of contaminating compounds (A260/A280 = 2.34 ± 0.41) compared to QIAamp^®^ DNA Stool Mini Kit (15.31 ± 18.78 ng/µL, A260/A280 = 1.98 ± 0.17). Moreover, all extracted samples gave positive results using the universal bacterial qPCR (the average Ct for all samples was 19.92 ± 3.97) indicating the absence of PCR inhibiters.

**Fig. 2:**
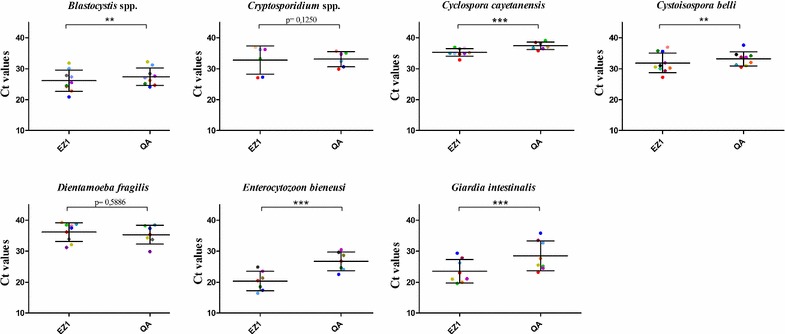
Ct values dot plots of PCR-positive samples for the seven eukaryotic enteric pathogens: *Blastocystis* spp. (n = 9), *Cryptosporidium parvum*/*hominis* (n = 8), *Cyclospora cayetanensis* (n = 10), *Dientamoeba fragilis* (n = 9), *Giardia intestinalis* (n = 8), *Cystoisospora belli* (n = 10) and *Enterocytozoon bieneusi* (n = 7) obtained with the two extraction procedures evaluated in the present study: EZ1^®^ (EZ1) and QIAamp^®^ DNA Stool Mini Kit^®^ (QA). Mean values and standard deviation ranges for each pathogen are represented by large and short horizontal bars, respectively. Statistical significance is represented as **(p < 0.002) and ***(p < 0.0001). Different colours indicate different samples. Same samples are represented by the same colour

The qPCR Ct values generated for each DNA template obtained with each extraction method is a function of the initial amount of parasite DNA in the amplification reactions. We use the Ct values as a surrogate marker of DNA purification efficiency. Each positive sample was extracted at the same time with these two methods, and qPCR detection was performed in the same CFX96™ run. No negative controls were amplified in any experiments. Figure 2 shows the Ct value distribution in PCR-positive samples obtained with both commercial methods. For *C. cayetanensis*, *G. intestinalis* and *E. bieneusi*, the Cts were significantly lower (p < 0.0001) with the EZ1^®^ compared to the QIAamp^®^ DNA Stool Mini Kit. Similarly, the Cts were significantly lower (p < 0.002) using the EZ1^®^ kit for *Blastocystis* spp. and *C. belli*. In contrast, there was no statistically significant difference between either method for the detection of *Cryptosporidium* spp. and *D. fragilis* DNA.

### Conclusion

A universal, reproducible, simple, efficient and robust extraction method is particularly valuable for PCR-based diagnosis which is increasingly used in both clinical laboratories and epidemiological studies [9, 12, 13]. To date, the EZ1^®^ procedure has been validated for the DNA extraction of viruses and bacteria [14] but also for fastidious microorganisms such as archaea [15]. Therefore, and in line with our findings, we recommend using the EZ1^®^ kit-based procedure, as described herein, for the PCR-based detection of eukaryotic intestinal pathogens in stool samples.

## Limitations

The main limitation of this study is the sample size that may have been too small due to the low number of positive stools in France. In fact, this work was carried out as a part of an epidemiological survey in order to select the most suitable extraction kit; the positive samples were harvested prospectively, which is why they are in limited numbers. Moreover, in this study, we focused on the eukaryotic enteric pathogens most found in France. In further investigations, it would be useful to test a larger number of eukaryotic enteric pathogens.

## Abbreviations

qPCR: quantitative PCR
Ct: cycle threshold
A260: the spectrophotometric absorbance at 260 nm
A260/A280: the ratio of spectrophotometric absorbance of each extracted sample at 260 nm to that of 280 nm
UDG: uracil-DNA glycosylase
